# Machine learning in the prediction of cardiac surgery associated acute kidney injury with early postoperative biomarkers

**DOI:** 10.3389/fsurg.2023.1048431

**Published:** 2023-02-07

**Authors:** Rui Fan, Wei Qin, Hao Zhang, Lichun Guan, Wuwei Wang, Jian Li, Wen Chen, Fuhua Huang, Hang Zhang, Xin Chen

**Affiliations:** ^1^ School of Medicine, Southeast University, Nanjing, China; ^2^ Department of Thoracic and Cardiovascular Surgery, Nanjing First Hospital, Nanjing Medical University, Nanjing, China; ^3^ Department of Nephrology, Nanjing First Hospital, Nanjing Medical University, Nanjing, China; ^4^ Department of Thoracic Surgery, Shanghai General Hospital, Shanghai Jiao Tong University School of Medicine, Shanghai, China

**Keywords:** acute kidney injury, cardiac surgery, biomarker, nomogram, machine learning, random forest

## Abstract

**Purpose:**

To establish novel prediction models for predicting acute kidney injury (AKI) after cardiac surgery based on early postoperative biomarkers.

**Patients and methods:**

This study enrolled patients who underwent cardiac surgery in a Chinese tertiary cardiac center and consisted of a discovery cohort (*n* = 452, from November 2018 to June 2019) and a validation cohort (*n* = 326, from December 2019 to May 2020). 43 biomarkers were screened using the least absolute shrinkage and selection operator and logistic regression to construct a nomogram model. Three tree-based machine learning models were also established: eXtreme Gradient Boosting (XGBoost), random forest (RF) and deep forest (DF). Model performance was accessed using area under the receiver operating characteristic curve (AUC). AKI was defined according to the Kidney Disease Improving Global Outcomes criteria.

**Results:**

Five biomarkers were identified as independent predictors of AKI and were included in the nomogram: soluble ST2 (sST2), N terminal pro-brain natriuretic peptide (NT-proBNP), heart-type fatty acid binding protein (H-FABP), lactic dehydrogenase (LDH), and uric acid (UA). In the validation cohort, the nomogram achieved good discrimination, with AUC of 0.834. The machine learning models also exhibited adequate discrimination, with AUC of 0.856, 0.850, and 0.836 for DF, RF, and XGBoost, respectively. Both nomogram and machine learning models had well calibrated. The AUC of sST2, NT-proBNP, H-FABP, LDH, and UA to discriminate AKI were 0.670, 0.713, 0.725, 0.704, and 0.749, respectively. In addition, all of these biomarkers were significantly correlated with AKI after adjusting clinical confounders (odds ratio and 95% confidence interval of the third vs. the first tertile: sST2, 3.55 [2.34–5.49], NT-proBNP, 5.50 [3.54–8.71], H-FABP, 6.64 [4.11–11.06], LDH, 7.47 [4.54–12.64], and UA, 8.93 [5.46–15.06]).

**Conclusion:**

Our study provides a series of novel predictive models and five biomarkers for enhancing the risk stratification of AKI after cardiac surgery.

## Introduction

Over the past few decades, advances in surgical techniques, anesthesia, and perioperative care have all contribute to enhancing in-hospital survival for cardiac patients. However, surgical morbidity is still a leading health burden in both developed and developing countries. As one of the most serious complications, cardiac surgery-associated acute kidney injury (CSA-AKI) was reported with the rate ranging 26.0%–28.5% (1). CSA-AKI not only severely affects acute morbidity and mortality, but also affects long-term prognosis.

Currently, because of a general lack of effective treatments for AKI, early identification of the condition is an important step to minimize the incidences of AKI and its mediated adverse events. Several attempts have been made to develop AKI prediction scores such as Cleveland Clinic score, Simplified Renal Index score, and Mehta score (2–4). However, these models were mainly derived based on traditional clinical factors from a patient's history (e.g., age, hypertension, diabetes mellitus, baseline kidney function), which provided limited information for AKI classification. On the other hand, due to differences in demographic characteristics and comorbidities, widely adoption of these models to other races or populations is of great challenge (5, 6).

Biomarkers have been proposed to clinical practice for more than 10 years. Unfortunately, serum creatinine (Scr), the main biomarker of AKI, does not increase until 50% of the kidney function is lost, potentially leading to diagnosis and treatment delay. Many novel biomarkers were proposed to substitute Scr in the assessment of kidney function, such as NGAL, KIM-1, L-FABP, and IGFBP-7 (7). However, in practice, a single biomarker is insensitive in its prediction for AKI. Therefore, it is necessary to consider a more comprehensive biomarker-based model to improve the accuracy and robustness of AKI prediction. The early postoperative period has important clinical implications as this time period is the optimal actionable window to optimize the management of postoperative AKI. Some of biomarkers arose during this period may reflect acute physiological responses for kidney function. In this study, we aimed to develop two types of models for CSA-AKI based on early postoperative biomarkers, applying multivariate logistic regression method and machine learning (ML) algorithms. The models were developed using a dataset of 452 patients from Nanjing First Hospital.

## Material and methods

### Study design and participants

This study contains two separate patient cohorts from our cardiac center. For the discovery cohort, participants admitted between November 2018 and June 2019 were retrospectively obtained from the Patient Information Management Platform of Nanjing First Hospital (218.2.200.37:2356/PatientList). This is a population-based database that consisted of patient information available from electronic health record in digital format. The clinical characteristics of patients were recorded in real time by medical personnel. The prediction models generated from the discovery stage were further validated in an independent patient cohort, which were prospectively enrolled between December 2019 and May 2020. This study was approved by the Ethical Committee of Nanjing First Hospital and informed consent was obtained from patients or their legal representatives. This study followed the TRIPOD statement guidelines for reporting (8).

We enrolled patients who underwent cardiac surgery with cardiopulmonary bypass (CPB) including coronary artery bypass grafting (CABG), valve surgery, and concomitant CABG and valve surgery. Patients were excluded if they met any of the following criteria: (i) aged <18 years old; (ii) AKI, dialysis, or end-stage renal disease at or before admission; (iii) emergency surgery; (iv) preoperative acute heart failure or hemodynamic instability; (v) missing data of Scr.

### Sample test

Laboratory biomarkers included cardiac biomarkers, arterial blood gas, blood biochemistry and blood cell analysis, and coagulation function. Arterial blood gases were collected immediately after intensive care unit (ICU) admission. In our center, most elective surgery can be done before 18.00 pm. Fasting venous blood samples were obtained on the morning (6:00 am) of the first postoperative day to assay cardiac biomarkers, blood biochemistry and blood cells, and coagulation function. When patients were in ICU, blood samples were collected for assessment of renal function around 6.00–7.00 am every day. When patients were transformed into normal ward, blood samples were collected for assessment of renal function around 8.00–9.00 am. All clinical samples were tested in the department of medical laboratory of Nanjing First Hospital.

### End point definition

The primary outcome was postoperative any-stage AKI, which was defined according to the Kidney Disease Improving Global Outcomes (KDIGO) clinical guideline (9), specifically an acute increase in Scr over 50% within 7 days, or 26.5 μmol/l elevation within 48 h compared with the reference Scr, or presence of oliguria (urine output less than 0.5 ml/kg/hr for 6 h), or a requirement of renal replacement therapy (RRT). The Scr levels measured before surgery were used as the reference value. Estimated glomerular filtration rate (eGFR) was calculated using the Chronic Kidney Disease Epidemiology Collaboration (CKD-EPI) creatinine equation (10).

### Model development

We developed a series of prediction models for CSA-AKI including a nomogram model and three tree-based ML models. Before developing the nomogram, we applied least absolute shrinkage and selection operator (LASSO) regression to identify a set of important biomarkers. The LASSO is a compressed estimation method based on the idea of data dimension reduction. It achieves the goal of variable selection by constructing a penalized function (*λ*) that compresses coefficients of irrelevant prediction variables towards zero (11). A particular advantage of this technique is that it avoids both overfitting and overestimation during model derivation. The tuning parameter (*λ*) was determined in the LASSO using 10-fold cross-validation on the basis of minimum criteria and a standard error. The identified biomarkers were then incorporated in a multivariate logistic regression analysis to generate a nomogram model.

We performed the following tree-based ML algorithms to develop the prediction models, which were the most popular and advanced ML methods used for the problem of classification, including eXtreme Gradient Boosting (XGBoost), RF, and deep forest (DF). XGBoost is a learning framework mainly consisting of two parts: simple decision tree and gradient boosting algorithm. XGBoost applies a second Taylor expansion on the loss function and simultaneously implements the first derivative and the second derivative (12). RF is a classic tree-based algorithm which combines multiple decision trees through majority voting (13). Both XGBoost and RF are ensemble learning methods characterized by good accuracy, robustness, and high calculating efficiency. As a more advanced deep learning approach, DF generates a multi-layer cascade forest, a structure contains many different RFs. The purpose of this design is to include different types of forests to ensure the diversity of the model. Each layer in the cascade forest receives the information processed by the previous stage and then concatenates with the original vector to be input to the next layer (14). (Supplementary Figure S1) To correctly interpret a ML model, we used Shapley Additive exPlanation (SHAP) values to explain the complex relationship between variables and outcomes in the RF model. The SHAP algorithm works based on the concept of Shapley values used in game theory (15). It provides a method to estimate the positive or negative contribution of individual feature to the overall model prediction *via* the GradientSHAP approximation (16).

### Statistical analysis

Continuous variables are presented as means ± standard deviations or medians (interquartile range), and categorized variables as frequencies with proportions. Comparisons between non-AKI group and AKI group were carried out using the t-test, Mann-Whitney U-test, chi-square test, or Fisher's exact probability method as appropriate. All demographic characteristics and clinical outcomes were available after a second manual review of the medical records. For laboratory data, our dataset has missing values ranging 0–3.8%. The missing data were handled using multiple imputation method. All laboratory biomarkers were log_e_ transformed for subsequent analysis.

The discrimination of prediction model was evaluated by area under the receiver operating characteristic (AUC). Model calibration was accessed using Brier score, and visualized with a 1000-resample bootstrapped calibration plot. A lower Brier score indicates superior model calibration (17). For interpreting ML model, we used SHAP summary plot to show feature importance and depicted the effect of specific feature on model output.

To further explore the association between single biomarker and AKI, we used the violin plots to show the distribution of the single biomarker among non-AKI and AKI groups. Receiver operating characteristic (ROC) curves were also formulated to determine the discriminative ability and optimal cut-off values of each biomarker (identified by the maximum Youden index). In addition, patients were divided into three group based on the tertiles of biomarker concentration distribution. Multivariate logistic regression analysis was performed to identify whether these biomarkers were predictors of AKI, independent of clinical confounders. Odds ratios (ORs) and corresponding two-sided 95% confidence intervals (CIs) were reported. Statistical analyses were performed using R (version 4.0.3) with the packages of *mice*, *glmnet*, and *rms*, and Python (version 3.8) with the packages of *sklearn*, *deep-forest*, and *shap*. A two-sided *P* value less than 0.05 indicated statistically significant.

## Results

### Characteristics of the cohorts

**Figure 1:**
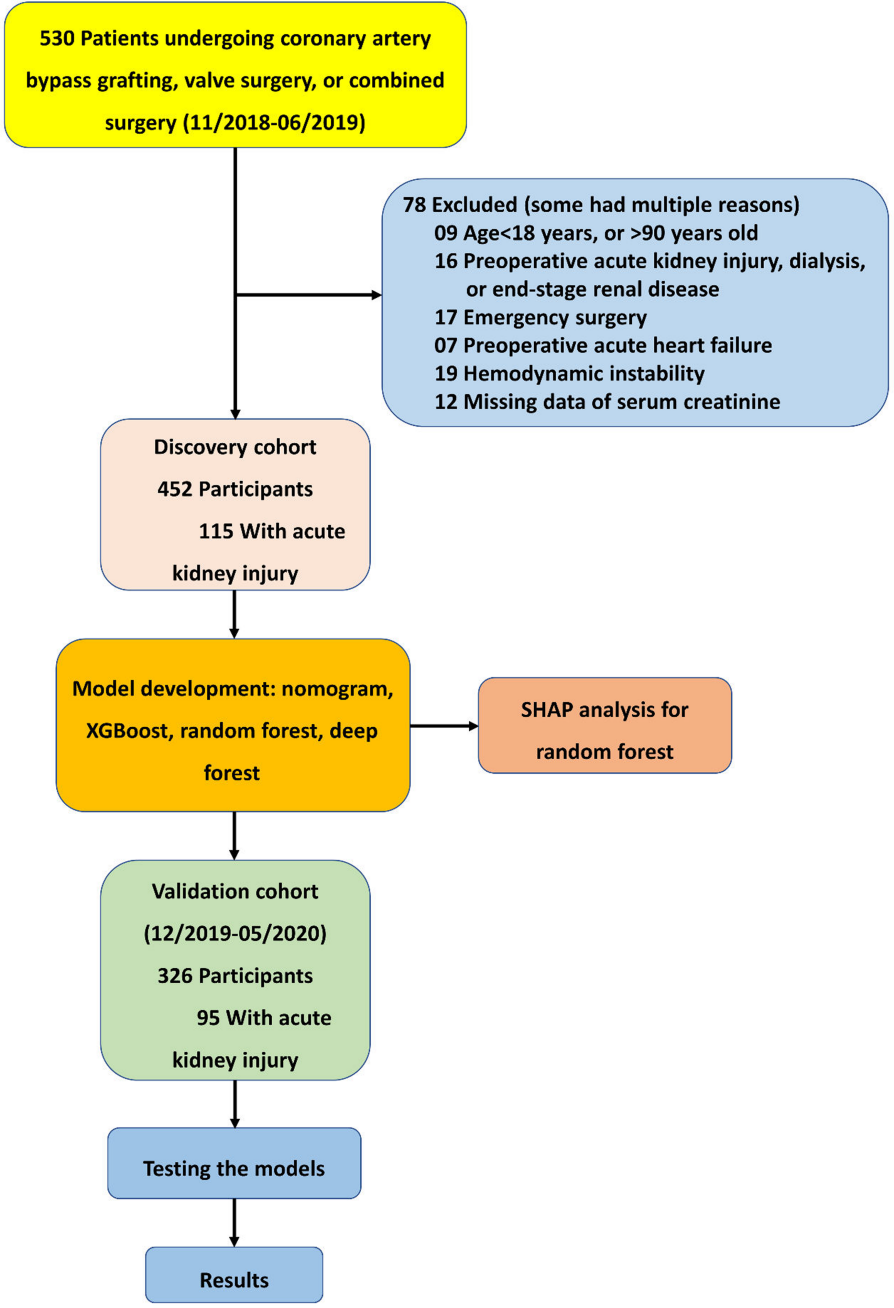
Formation of the discovery and validation cohorts of acute kidney injury after cardiac surgery.

Overall, 452 participants, admitted between November 2018 and June 2019, comprised the discovery cohort; 326 participants, admitted between December 2019 and May 2020, comprised the validation cohort (Figure 1). The rates of AKI were 25.4% and 29.1% in the discovery and validation cohorts, respectively. In the discovery cohort, patients who developed AKI showed a significant association on the univariate analysis included advanced age (*P* = 0.009), more diabetes mellitus (*P* = 0.038), hypertension (*P* = 0.047), and combined surgery (*P* = 0.008), longer duration of CPB and aortic cross-clamping (all *P* < 0.001). Patients who developed AKI had more death or on RRT (*P* = 0.044), more pulmonary infection (*P* = 0.004), longer ventilation time, ICU stay and hospital stay (all *P* < 0.001). Similar differences were observed in the validation cohort except diabetes mellitus (*P* = 0.440), hypertension (*P* = 0.403), and death or on RRT (*P* = 0.496) (Table 1).

**Table 1:** Clinical characteristics of patients in the discovery and validation cohorts who did or did not develop acute kidney injury.

Characteristics	Discovery cohort (*n* = 452)		*P*-value	Validation cohort (*n* = 326)		*P*-value
	Non-AKI (*n* = 337)	AKI (*n* = 115)		Non-AKI (*n* = 231)	AKI (*n* = 95)	
Age (years)	61.8 ± 9.9	64.1 ± 10.7	0.009	60.5 ± 11.2	65.2 ± 9.3	<0.001
Male	202 (59.9)	72 (62.6)	0.613	141 (61.0)	60 (63.2)	0.721
Body mass index (kg/m^2^)	24.0 ± 3.29	24.3 ± 3.21	0.453	23.8 ± 3.23	24.0 ± 3.44	0.672
Diabetes mellitus	63 (18.7)	32 (27.8)	0.038	47 (20.3)	23 (24.2)	0.440
Hypertension	184 (54.6)	75 (65.2)	0.047	122 (52.8)	55 (57.9)	0.403
eGFR (ml/min/1.73 m^2^)	96.2 (24.8)	90.2 (31.7)	<0.001	102.4 (30.9)	86.8 (30.1)	<0.001
COPD	15 (4.5)	5 (4.4)	0.963	10 (4.3)	5 (5.3)	0.714
Cerebrovascular accident	30 (8.9)	9 (7.8)	0.723	26 (11.3)	10 (10.5)	0.849
LVEF	62.0 (58.0–64.0)	61.0 (53.0–64.0)	0.052	62.0 (58.0–64.0)	62.0 (56.0–65.0)	0.722
NYHA III-IV	78 (23.1)	33 (28.7)	0.232	62 (26.8)	30 (31.6)	0.388
Prior myocardial infarction	30 (8.9)	14 (12.2)	0.307	27 (11.7)	14 (14.7)	0.812
Surgery type			0.008			0.024
CABG alone	130 (38.6)	33 (28.7)		75 (32.5)	23 (24.2)	
Valve surgery alone	172 (51.0)	58 (50.4)		134 (58.0)	53 (55.8)	
CABG and valve surgery	35 (10.4)	24 (20.9)		22 (9.5)	19 (20.0)	
CPB time (min)	91.0 (71.0–115)	113.0 (87.0–142.0)	<0.001	97.0 (81.0–126.0)	111.0 (94.5–133.0)	0.002
ACC time (min)	63.0 (46.0–83.0)	74.0 (56.5–97.0)	<0.001	68.0 (52.5–89.5)	76.0 (62.0–91.5)	0.015
**In-hospital outcome**						
Death or on RRT	3 (0.9)	5 (4.3)	0.044	3 (1.3)	3 (3.2)	0.496
Acute myocardial infarction	1 (0.3)	1 (0.9)	1.000	1 (0.4)	0 (0)	1.000
Acute heart failure	3 (0.9)	3 (0.9)	0.358	1 (0.4)	2 (2.1)	0.424
Hepatic insufficiency	3 (0.9)	4 (3.5)	0.133	3 (1.3)	2 (2.1)	0.966
Pulmonary infection	7 (2.1)	9 (7.8)	0.004	4 (1.7)	7 (7.4)	0.026
Reoperation for bleeding	2 (0.6)	3 (2.6)	0.205	4 (1.7)	5 (5.2)	0.163
Ventilation time (hr)	13.0 (12.0–18.0)	16.0 (13.0–24.0)	<0.001	14.0 (12.0–20.0)	17.0 (14.0–22.0)	0.001
ICU length of stay (hr)	23.0 (21.0–43.0)	42.0 (21.5–47.0)	<0.001	24.0 (21.0–44.0)	40.0 (21.0–57.0)	0.005
Hospital length of stay (d)	16.0 (13.0–19.0)	19.0 (15.0–24.0)	<0.001	16.0 (14.0–20.0)	19.0 (15.5–23.0)	<0.001

Data are reported as mean ± SD, medians (interquartile range), or percentage values. AKI, acute kidney injury; eGFR, estimated glomerular filtration rate; COPD, chronic obstructive pulmonary disease; LVEF, left ventricular ejection fraction; NYHA, New York Heart Association; CABG, coronary artery bypass grafting; CPB, cardiopulmonary bypass; ACC, aortic cross-clamping; RRT, renal replacement therapy; ICU, intensive care unit.

43 early postoperative biomarkers were summarized in the discovery cohort (Table 2). Patients who developed AKI had higher indices of soluble ST2 (sST2), N terminal pro-brain natriuretic peptide (NT-proBNP), heart-type fatty acid binding protein (H-FABP), aspartate transaminase, lactic dehydrogenase (LDH), uric acid (UA), total bile acid, serum potassium, serum magnesium, or had lower indices of high-density lipoprotein (HDL), PO_2_, hemoglobin, hematocrit (all *P* < 0.05).

**Table 2:** Early postoperative biomarkers among patients in the discovery cohort who did or did not develop acute kidney injury.

Biomarker	Total (*n* = 452)	Non-AKI (*n* = 337)	AKI (*n* = 115)	*P*-value
sST2	4. q 67 ± 1.20	4.51 ± 1.16	5.13 ± 1.18	<0.001
NT-proBNP	6.35 ± 1.16	6.11 ± 1.00	7.03 ± 1.31	<0.001
H-FABP	1.27 ± 1.28	1.00 ± 1.23	2.06 ± 1.09	<0.001
**Biochemical test**				
ALT	3.16 ± 0.63	3.18 ± 0.63	3.09 ± 0.62	0.156
AST	3.92 ± 0.58	3.87 ± 0.57	4.07 ± 0.59	0.002
LDH	5.83 ± 0.33	5.78 ± 0.28	5.98 ± 0.41	<0.001
Alkaline phosphatase	3.88 ± 0.49	3.86 ± 0.47	3.92 ± 0.55	0.270
UA	5.66 ± 0.33	5.59 ± 0.32	5.87 ± 0.28	<0.001
Total cholesterol	0.98 ± 0.27	0.99 ± 0.26	0.96 ± 0.29	0.319
Triglyceride	0.06 ± 0.44	0.06 ± 0.43	0.07 ± 0.47	0.945
HDL	−0.30 ± 0.25	−0.28 ± 0.24	−0.35 ± 0.27	0.007
LDL	0.32 ± 0.39	0.33 ± 0.38	0.30 ± 0.41	0.466
Apolipoprotein A1	−0.14 ± 0.22	−0.13 ± 0.21	−0.17 ± 0.22	0.054
Apolipoprotein B	−0.74 ± 0.33	−0.74 ± 0.32	−0.75 ± 0.36	0.680
Lipoprotein (a)	4.79 ± 0.86	4.78 ± 0.86	4.81 ± 0.86	0.721
Serum albumin	3.50 ± 0.12	3.50 ± 0.12	3.50 ± 0.12	0.739
Total bilirubin	2.69 ± 0.53	2.69 ± 0.52	2.66 ± 0.56	0.605
TBA	−0.50 ± 0.91	−0.56 ± 0.88	−0.31 ± 0.95	0.014
**Arterial blood gas**				
PCO_2_	3.44 ± 0.17	3.44 ± 0.17	3.45 ± 0.17	0.823
PO_2_	4.97 ± 0.35	4.99 ± 0.34	4.91 ± 0.38	0.040
Intubated PO_2_/FiO_2_ ratio	5.96 ± 0.37	5.89 ± 0.35	5.78 ± 0.41	0.013
Serum sodium	4.94 ± 0.06	4.94 ± 0.07	4.94 ± 0.02	0.489
Serum potassium	1.46 ± 0.15	1.44 ± 0.14	1.49 ± 0.16	0.003
Serum calcium	0.16 ± 0.05	0.16 ± 0.05	0.16 ± 0.05	0.348
Serum magnesium	−0.50 ± 0.19	−0.52 ± 0.19	−0.46 ± 0.18	0.005
Lactic acid	0.48 ± 0.60	0.47 ± 0.60	0.52 ± 0.61	0.447
HCO_3−_	2.98 ± 0.22	2.98 ± 0.24	2.98 ± 0.12	0.790
**Blood cell analysis**				
WBC count	2.57 ± 0.28	2.57 ± 0.28	2.58 ± 0.29	0.705
Lymphocyte count	−0.39 ± 0.49	−0.39 ± 0.50	−0.41 ± 0.47	0.662
Monocyte count	−0.14 ± 0.48	−0.14 ± 0.46	−0.14 ± 0.53	0.979
Neutrophil count	2.43 ± 0.29	2.43 ± 0.29	2.44 ± 0.30	0.663
RBC count	1.25 ± 0.14	1.25 ± 0.14	1.23 ± 0.15	0.129
Hemoglobin	4.66 ± 0.13	4.67 ± 0.13	4.64 ± 0.15	0.025
Hematocrit	3.46 ± 0.13	3.46 ± 0.13	3.43 ± 0.14	0.045
RDW	2.61 ± 0.11	2.60 ± 0.11	2.63 ± 0.12	0.061
Platelet count	4.96 ± 0.42	4.96 ± 0.39	4.95 ± 0.50	0.783
MPV	2.46 ± 0.10	2.46 ± 0.10	2.46 ± 0.08	0.890
PDW	2.67 ± 0.17	2.67 ± 0.18	2.68 ± 0.14	0.881
**Coagulation function**				
PT	2.46 ± 0.14	2.45 ± 0.15	2.47 ± 0.09	0.102
INR	0.02 ± 0.10	0.02 ± 0.09	0.04 ± 0.12	0.064
APTT	3.30 ± 0.21	3.30 ± 0.23	3.31 ± 0.14	0.352
Fibrinogen	1.03 ± 0.29	1.03 ± 0.29	1.05 ± 0.29	0.561
D-Dimer	−0.13 ± 0.70	−0.11 ± 0.69	−0.19 ± 0.75	0.333

All variables are log_e_ transformed and presented as mean ± SD. AKI, acute kidney injury; sST2, soluble ST2; NT-proBNP, N terminal pro-brain natriuretic peptide; H-FABP, heart-type fatty acid-binding protein; ALT, alanine aminotransferase; AST, aspartate transaminase; LDH, lactic dehydrogenase; UA, uric acid; HDL, high density lipoprotein; LDL, low density lipoprotein; TBA, total bile acid; WBC, white blood cell; RBC, red blood cell; RDW, red blood cell distribution width; MPV, mean platelet volume; PDW, platelet distribution width; PT, prothrombin time; INR, international normalized ratio; APTT, activated partial thromboplastin time.

### Variable selection

In the discovery cohort, 43 biomarkers were included in the variable selection procedure. The LASSO identified six biomarkers predisposing to AKI: sST2, NT-proBNP, H-FABP, LDH, UA, and HDL (Supplementary Figure S2). Inclusion of these six variables in a logistic regression model resulted in five variables (excluded HDL, *P* > 0.05) that were independently statistically significant predictors of AKI and were included in the final model (Table 3).

**Table 3:** Multivariate logistic regression model for predicting acute kidney injury based on discovery cohort.

Risk factor	*β*	OR (95% CI)	*P*-value
sST2	0.407	1.50 (1.18–1.91)	0.001
NT-proBNP	0.706	2.03 (1.58–2.59)	<0.001
H-FABP	0.784	2.19 (1.71–2.80)	<0.001
LDH	0.964	2.62 (1.09–6.31)	0.031
UA	2.789	16.27 (5.31–49.82)	<0.001
Intercept	−30.489		

Calculation of predicted risk using patient data and β regression coefficients: Calculate the odds of acute kidney injury = exp (−30.4898 + [0.407 × log_e_ sST2 ng/ml] + [0.706 × log_e_ NT-proBNP pg/ml] + [0.784 × log_e_ H-FABP ng/ml] + [0.964 × log_e_ LDH u/l] + [2.789 × log_e_ UA umol/l]).

Predicted risk of CSA-AKI as a percentage = [odds/(1 + odds)] × 100.

OR, odds ratio; sST2, soluble ST2; NT-proBNP, N terminal pro-brain natriuretic peptide; H-FABP, heart-type fatty acid-binding protein; LDH, lactic dehydrogenase; UA, uric acid.

### Nomogram and model performance

**Figure 2:**
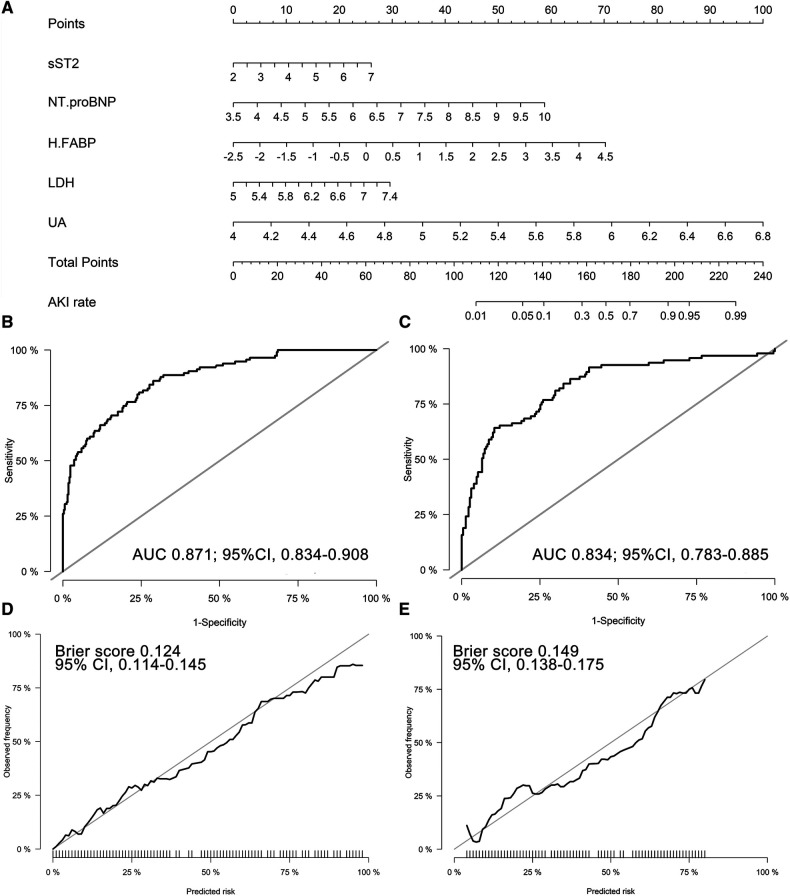
Biomarker-based nomogram model and model performance. (**A**) Nomogram to predict CSA-AKI; Receiver operating characteristic curve of the nomogram in the discovery (**B**) and validation (**C**) cohorts; 1000-resample bootstrapped calibration plot of the nomogram in the discovery (**D**) and validation (**E**) cohorts. sST2, soluble ST2; NT-proBNP, N terminal pro-brain natriuretic peptide; H-FABP, heart-type fatty acid-binding protein; LDH, lactic dehydrogenase; UA, uric acid; AUC, area under the receiver operating characteristic curve.

An AKI prediction nomogram model was constructed (Figure 2A). The nomogram demonstrated good discrimination in the discovery cohort, with a C-statistic of 0.871 (95%CI 0.834–0.908). Correspondingly, in the validation cohort, the nomogram achieved a C-statistic of 0.834 (95%CI 0.783–0.885) (Figures 2B,C). The calibration plots revealed good calibration in both cohorts (Figures 2D,E).

### Ml models

**Figure 3:**
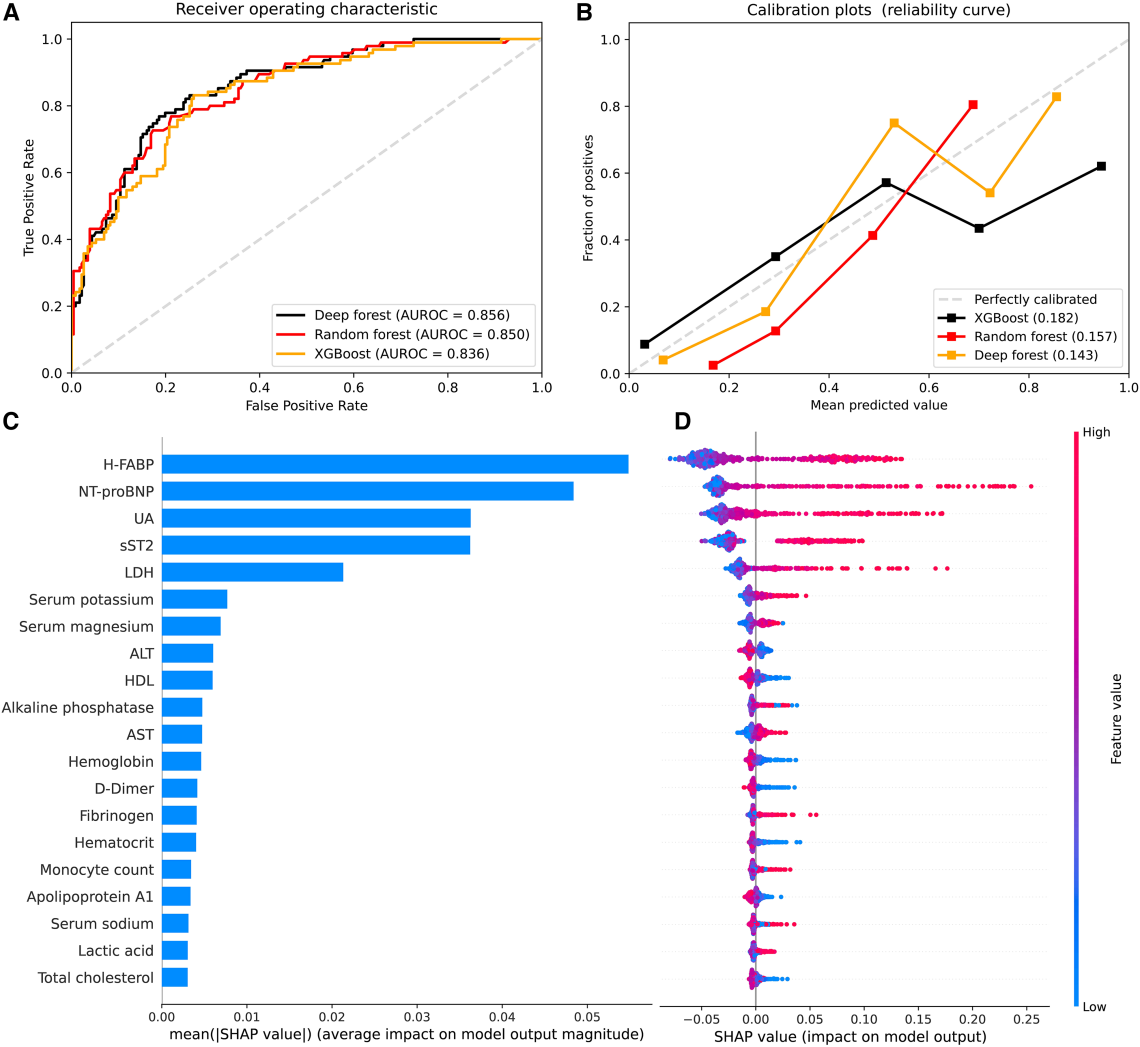
Model performance and SHAP analysis of machine learning models. (**A**) Receiver operating characteristic curve to assess discrimination of XGBoost, random forest, and deep forest models in the validation cohort; (**B**) Calibration plots for XGBoost, random forest, and deep forest models in the validation cohort (5 bins); (**C**) SHAP summary plots of top 20 features in the random forest. Feature importance ranked by SHAP values; (**D**) Dot estimation for each feature's attribution value to model output. The higher SHAP value of features, the higher risk of acute kidney injury. SHAP, Shapely Additive exPlanations; H-FABP, heart-type fatty acid-binding protein; NT-proBNP, N terminal pro-brain natriuretic peptide; sST2, soluble ST2; UA, uric acid; LDH, lactic dehydrogenase; HDL, high density lipoprotein; AST, aspartate transaminase; ALT, alanine aminotransferase; TBA, total bile acid.

We constructed ML models using XGBoost, RF, and DF algorithms with all the biomarkers as input variables. In the validation cohort, the AUCs were 0.856 (95%CI 0.813–0.899) for RF model, 0.850 (95%CI 0.805–0.894) for DF model, and 0.836 (95%CI 0.790–0.883) for XGBoost model (Figure 3A). Different accuracy metrics were displayed in Table 4. The DF model exhibited the best calibration (Brier score: 0.143), followed by RF (Brier score: 0.157) and XGBoost (Brier score: 0.182) (Figure 3B). The SHAP values were used to highlight individual contributions of the variables in the RF model. Figure 3C describes the SHAP summary plot, showing the SHAP values in order of the important variables that contribute to AKI. According to the summary plot, each dot represents one patient and the horizontal location of each dot indicates whether the effect of a feature is associated with a higher or lower risk of AKI (Figure 3D).

**Table 4:** Accuracy metrics of three machine models.

Model	Accuracy	Sensitivity (%)	Specificity (%)	PPV (%)	NPV (%)	F1 score
XGBoost	0.808	57.8	89.4	74.9	82.1	0.79
Random forest	0.812	56.9	87.5	76.2	84.2	0.79
Deep forest	0.819	60.1	90.0	76.9	84.4	0.83

PPV, positive predictive value; NPV, negative predictive value.

### Association between single biomarker and AKI

**Figure 4:**
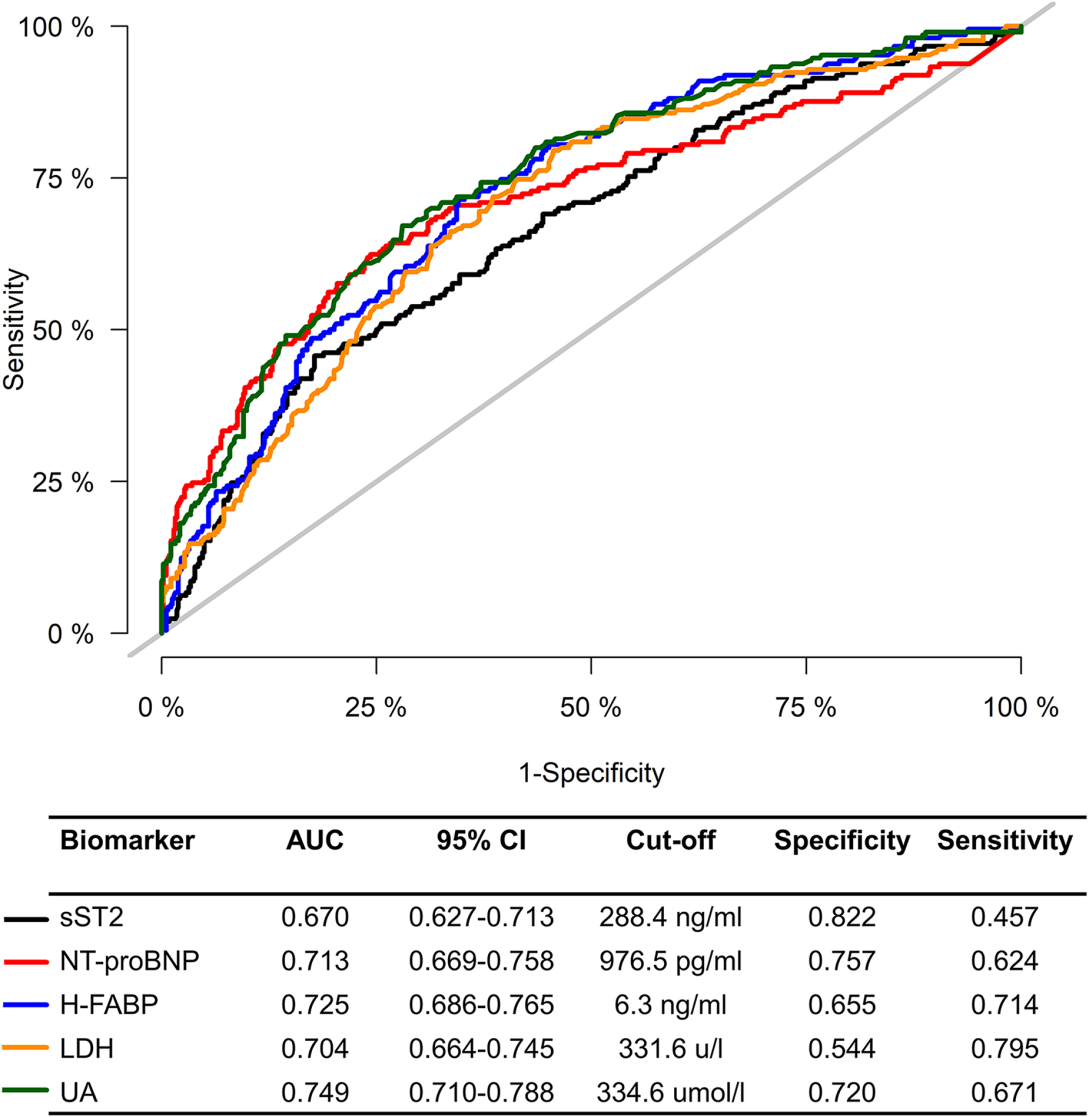
Receiver operating characteristic curve for sST2, NT-proBNP, H-FABP, LDH, and UA to predict CSA-AKI. AKI, acute kidney injury; AUC, area under the receiver operating characteristic curve; CI, confidence interval; sST2, soluble ST2; NT-proBNP, N terminal pro-brain natriuretic peptide; H-FABP, heart-type fatty acid-binding protein; LDH, lactic dehydrogenase; UA, uric acid.

We further investigated the association of sST2, NT-proBNP, H-FABP, LDH, and UA with AKI after pooling the two cohorts together. The violin plots showed that patients in the AKI group had significantly higher levels of sST2, NT-proBNP, H-FABP, LDH, and UA than patients without AKI (Supplementary Figure S3). ROC curves were used to test the overall discriminative ability of these five biomarkers for AKI. We observed that the AUCs of sST2, NT-proBNP, H-FABP, LDH, and UA to discriminate AKI were 0.670 (95%CI, 0.627–0.713), 0.713 (95%CI, 0.669–0.758), 0.725 (95%CI, 0.686–0.765), 0.704 (95%CI, 0.664–0.745), and 0.749 (95%CI, 0.710–0.788). Meanwhile, the optimal cut-off values and corresponding specificity and sensitivity were determined (Figure 4). Moreover, we divided patients into three groups based on the tertiles of biomarker concentration distribution. With the first tertile used as the reference category, increased levels of these five biomarkers were associated with a higher risk of AKI. These associations persisted after multifactorial adjustment, including baseline eGFR (Table 5).

**Table 5:** Multivariate regression analysis on the association between sST2, NT-proBNP, H-FABP, LDH, and UA with acute kidney injury in patients of the total cohort.

Biomarker	Range	Crude OR	*P* value	Adjust OR^a^	Adjust *P*-value^a^
**sST2**					
T1	2.25–4.31	1	Ref	1	Ref
T2	4.31–5.12	1.67 (1.08–2.61)	0.022	1.76 (1.12–2.77)	0.014
T3	5.13–6.75	3.79 (2.52–5.78)	<0.001	3.55 (2.34–5.49)	<0.001
**NT-proBNP**					
T1	3.57–5.93	1	Ref	1	Ref
T2	5.93–6.99	1.15 (0.73–1.83)	0.545	1.09 (0.68–1.77)	0.704
T3	7.01–9.76	5.44 (3.62–8.32)	<0.001	5.50 (3.54–8.71)	<0.001
**H-FABP**					
T1	-2.30–1.19	1	Ref	1	Ref
T2	1.20–2.24	3.19 (1.97–5.28)	<0.001	3.04 (1.86–5.09)	<0.001
T3	2.24–6.08	7.35 (4.64–12.01)	<0.001	6.64 (4.11–11.06)	<0.001
**LDH**					
T1	5.00–5.68	1	Ref	1	Ref
T2	5.69–5.96	2.65 (1.67–4.29)	<0.001	3.02 (1.85–5.04)	<0.001
T3	5.96–7.32	5.91 (3.80–9.42)	<0.001	7.47 (4.54–12.64)	<0.001
**UA**					
T1	2.05–5.58	1	Ref	1	Ref
T2	5.58–5.85	2.22 (1.37–3.68)	<0.001	2.40 (1.45–4.06)	0.001
T3	5.85–6.65	8.03 (5.13–12.95)	<0.001	8.93 (5.46–15.06)	<0.001

All biomarkers are calculated on log_e_-transformed. T1-T3 represents Tertile 1–3.

^a^ Adjusted multifactorially for age, male, body mass index, diabetes mellitus, hypertension, chronic obstructive pulmonary disease, cerebrovascular accident, left ventricular ejection fraction, New York Heart Association III-IV, combined surgery, and baseline estimated glomerular filtration rate. OR, odds ratio; sST2, soluble ST2; NT-proBNP, N terminal pro-brain natriuretic peptide; H-FABP, heart-type fatty acid-binding protein; LDH, lactic dehydrogenase; UA, uric acid.

## Discussion

In this study, using early postoperative biomarkers, we constructed a series of models for enhancing risk stratification of AKI after cardiac surgery. The discrimination of these models was clinically satisfactory, with AUCs ranging 0.834–0.856 in the validation cohort. In addition, both LASSO and SHAP analysis identified sST2, NT-proBNP, H-FABP, LDH, and UA as the most influential predictors of AKI.

To the best of our knowledge, the biomarker-based nomogram model in this study consists of four cardiac biomarkers (sST2, NT-proBNP, H-FABP, and LDH) and a metabolism related biomarker (UA), which have not been included in previous studies. Overall, they are novel biomarkers for predicting AKI. sST2 is initially found to be a member of the interleukin-1 (IL-1) receptor family that serves as a decoy for IL-33, regulating immune and inflammatory responses. Emerging evidence from epidemiological studies supported that elevated serum sST2 levels were associated with mortality and adverse clinical outcomes in patients suffering from heart failure, coronary artery disease, arrhythmia, and stroke (18–21). Lobdell et al. demonstrated a significant association of high preoperative concentrations of sST2 as a prognostic indicator of AKI among patients undergoing CABG (22). We observed a similar statistical difference in early postoperative phase. These findings may promote the extension of the predictive ability of sST2 from cardiovascular disease to kidney injury.

As an intracellular transport protein responsible for transporting free fatty acid in cardiomyocytes, H-FABP has been recognized as a diagnostic and prognostic marker for acute coronary syndrome (23, 24). Schaub et al. explored the predictive value of H-FABP for AKI at four time points perioperatively (25). They found that patients who developed AKI had higher H-FABP than those who did not. Published literatures gave plausible explanations of the relationship between H-FABP and AKI in cardiac patients (25–27). First, high H-FABP levels were only observed in patients who had received on-pump cardiac surgery, but not in off-pump procedures. This indicated that ischemia-reperfusion injury and inflammatory might play a role in the elevation of H-FABP. Second, H-FABP was an indicator of hemodynamic instability after cardiac surgery. Third, elevated H-FABP levels were also found in patients with elevated venous pressure or venous congestion, which in return would affect kidney circulation and lead to “congestive kidney failure”.

Like sST2 and H-FABP, NT-proBNP and LDH are also cardiac biomarkers associated with development of AKI. NT-proBNP is mainly cleared by renal excretion, and its change has a significant modifying effect on kidney function decline (28). Besides, postoperative cardiac insufficiency, which is predicted by NT-proBNP, promotes AKI *via* hypotension. LDH anomaly is known to often occur in certain diseases such as renal failure, cardiovascular events, and hepatic damage. Therefore, LDH was usually measured to detect tissue damage as well as patient's overall disease severity (29). Moreover, in the CPB setting, the elevated LDH in the immediate postoperative period may be an indicator of CPB-induced hemolysis, which is associated with the development of AKI (30). UA is the final product of endogenous and exogenous purine metabolism. A population-based cohort study has shown that high serum UA levels were positively associated with elevated levels of pro-inflammatory cytokines (e.g., interleukin-6, high sensitivity C-reactive protein, tumor necrosis factor-α), which was considered as the central components of the pathogenesis of AKI (31). Kidney function may benefit from UA lowering therapy; therefore, UA may serve as a novel potential target for AKI prevention (32).

The advantage of our study included the use of SHAP values to uncover the black box of MLthe DF algorithm to predict CSA-AKI. As one of the advanced tree-based learning methods, the DF model demonstrated better predictive power than the conventional ML model. The DF boosts predictive information by integrating multiple RFs, providing an effective option to investigate binary problem and improving the robustness of the standard deep learning methods working on small-scale data. Compared with logistic regression, machine learning can include much more variables and resolve nonlinear interactions. This can improve the effectiveness of prediction. Even though AUC of machine learning we used was 0.856, which was similar to the logistic regression, it was higher than AUC of logistic regression (0.834). This means machine learning showed better performance than traditional analysis. To feasibility, these biomarkers can be collected easily from venous blood and tested in labs easily.

Several limitations in this study should also be noted. First, the models were derived from a single-center dataset. Therefore, before models can be implemented in clinical practice, their predictive power needed to be validated in external datasets. Second, we did not consider innovative biomarkers such as renal tubule-associated biomarkers (e.g., NGAL, KM-1, MMP-7) or biomarkers of inflammation (e.g., IL-6, IL-10, and TNF-α), which may also be ideal predictors of AKI. But they remained poorly studied as a biomarker-based prediction tool in general or cardiac surgery. Third, we did not include traditional scoring systems (e.g., Cleveland Clinic score, Simplified Renal Index score, Mehta score) to make model comparisons because most of these models were specially designed to predict AKI requiring RRT. Given the high incidence of mild or moderate AKI and its strong association with adverse outcomes, more efforts should be made to predict any-stage AKI after cardiac surgery.

## Conclusion

We successfully constructed a nomogram and three tree-based ML models to predict CSA-AKI based on early postoperative biomarkers. Our study identified five important biomarkers (sST2, NT-proBNP, H-FABP, LDH, and UA) associated with CSA-AKI.

## Data Availability

The raw data supporting the conclusions of this article will be made available by the authors, without undue reservation.
