# Supplementary material for: Machine learning in the prediction of cardiac surgery associated acute kidney injury with early postoperative biomarkers
Source: Front Surg. 2023 Feb 7;10:1048431. doi: 10.3389/fsurg.2023.1048431 (PMC9942777; doi:10.3389/fsurg.2023.1048431)
Supplement: Supplementary file 1 [file Datasheet1.pdf]

Supplementary Materials

Supplementary Figure 1 Illustration of cascade forest structure.

Supplementary Figure 2 The result of LASSO in identifying biomarkers of predicting AKI in discovery cohort.

Supplementary Figure 3 The violin plot in the distribution of sST2, NT-proBNP, H-FABP, LDH, and UA among the non-AKI and AKI groups.

Supplementary Table 1 Early postoperative biomarkers among patients in the validation cohort who did or did not develop acute kidney injury.

Supplementary Table 2 Percentage of missing variables for each predictor in discovery cohort.

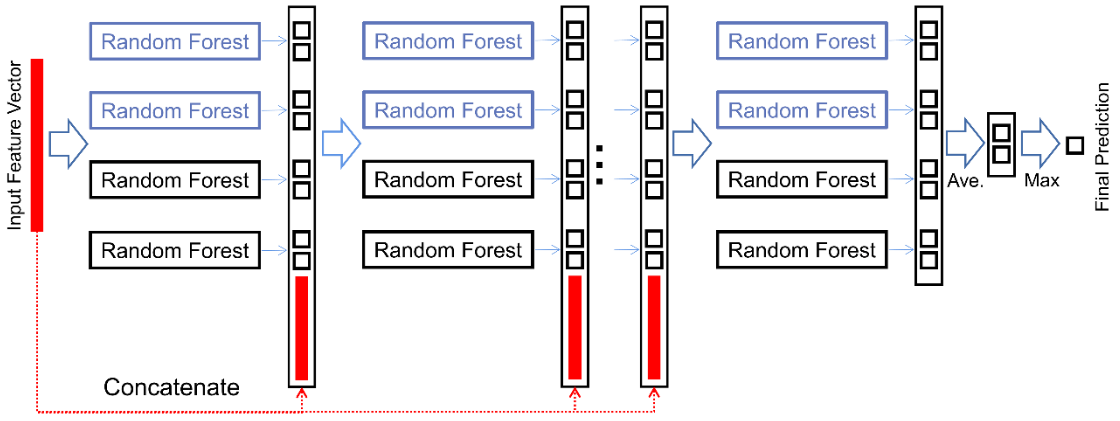

Supplementary Figure 1 Illustration of cascade forest structure. Each level of the cascade consists of two random forests (light blue) and two extra random forests (black). Each forest outputs a two-dimensional class vector, which is then concatenated for representation of original input.

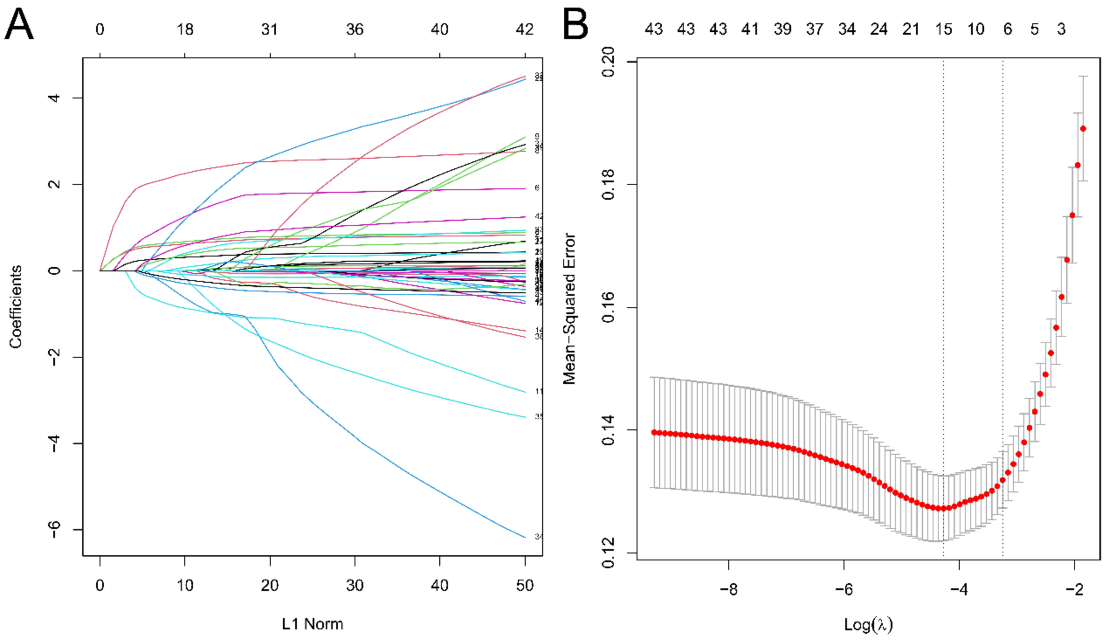

Supplementary Figure 2 The result of LASSO in identifying biomarkers of predicting AKI in discovery cohort. Variable selection using least absolute shrinkage and selection operator regression. (**A**) LASSO coefficient profiles of 43 variables. (**B**) Identification of optimal penalization coefficient (λ) using 10-fold cross-validation via optimal λ at minimum criteria and a standard error (six variables).

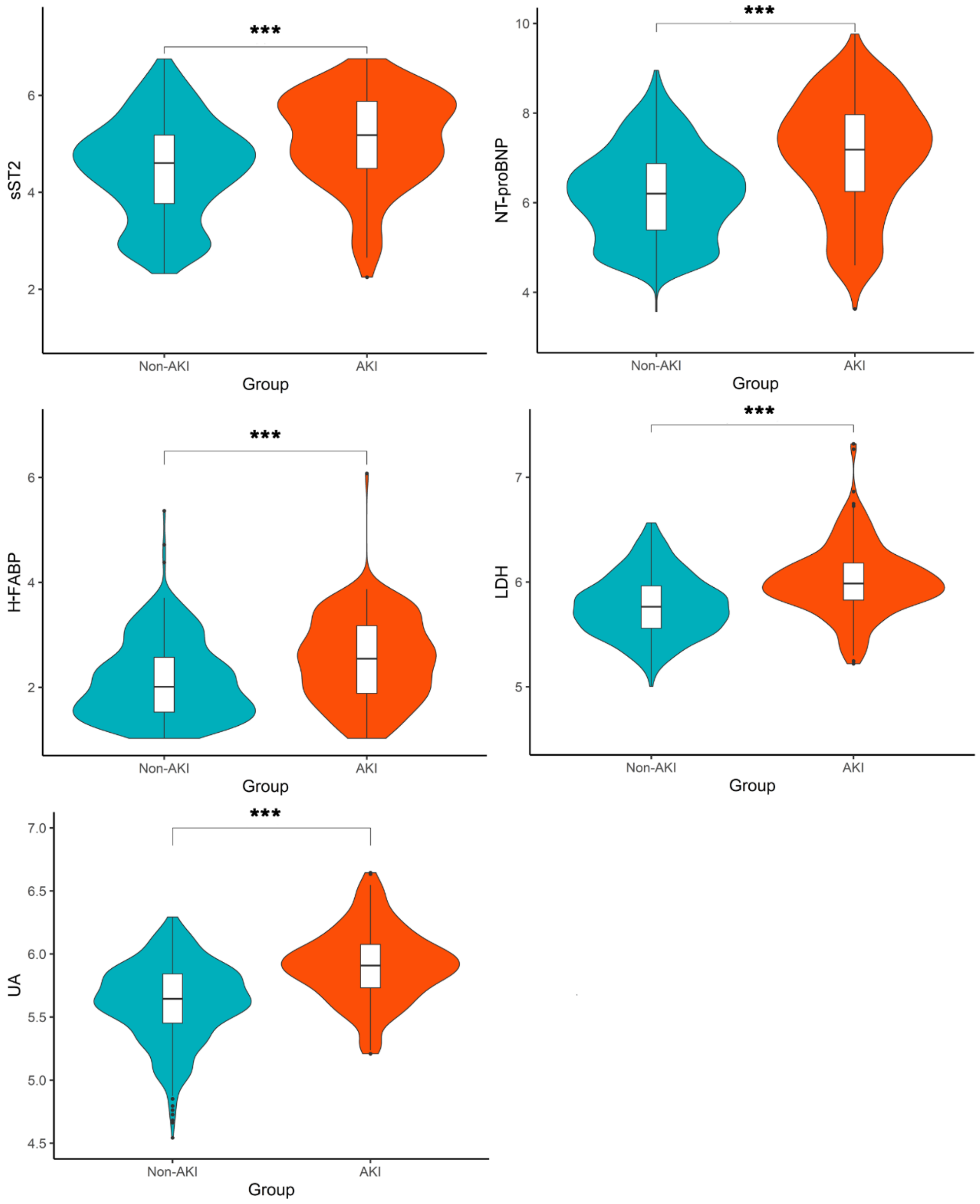

Supplementary Figure 3 The violin plot in the distribution of sST2, NT-proBNP, H-FABP, LDH, and UA among the non-AKI and AKI groups. Differences in biomarker concentrations were compared after pooling the two cohort together. Statistical significance was determined using the student's t-test (***P<0.001). AKI, acute kidney injury; sST2, soluble ST2; NT-proBNP, N terminal pro-brain natriuretic peptide; H-FABP, heart-type fatty acid-binding protein; LDH, lactic dehydrogenase; UA, uric acid.

Supplementary Table 1 Early postoperative biomarkers among patients in the validation cohort who did or did not develop acute kidney injury

**Biomarker**	**Total (n=326)**	**Non-AKI (n=231)**	**AKI (n=95)**	**P-value**
sST2	4.66 ± 0.82	4.42 ± 0.75	5.25 ± 0.68	<0.001
NT-proBNP	6.55 ± 1.13	6.32 ± 1.00	7.13 ± 1.21	<0.001
H-FABP	2.15 ± 0.87	2.03 ± 0.81	2.44 ± 0.94	<0.001
**Biochemical test**				
ALT	2.92 ± 0.62	2.94 ± 0.62	2.89 ± 0.63	0.544
AST	3.85 ± 0.65	3.80 ± 0.64	3.99 ± 0.67	0.016
LDH	5.85 ± 0.31	5.78 ± 0.32	6.05 ± 0.21	<0.001
Alkaline phosphatase	3.98 ± 0.32	3.97 ± 0.30	4.01 ± 0.36	0.353
Uric acid	5.73 ± 0.41	5.65 ± 0.32	5.91 ± 0.53	<0.001
Total cholesterol	1.03 ± 0.29	1.04 ± 0.27	1.02 ± 0.32	0.677
Triglyceride	0.14 ± 0.42	0.12 ± 0.41	0.16 ± 0.44	0.441
HDL	-0.38 ± 0.29	-0.36 ± 0.29	-0.43 ± 0.29	0.038
LDL	0.35 ± 0.39	0.36 ± 0.38	0.34 ± 0.44	0.741
Apolipoprotein A1	-0.20 ± 0.19	-0.19 ± 0.19	-0.21 ± 0.20	0.450
Apolipoprotein B	-0.75 ± 0.36	-0.75 ± 0.34	-0.74 ± 0.40	0.732
Lipoprotein (a)	4.89 ± 0.79	4.84 ± 0.77	5.04 ± 0.83	0.043
Serum albumin	3.50 ± 0.23	3.51 ± 0.25	3.47 ± 0.16	0.093
Total bilirubin	2.58 ± 0.48	2.61 ± 0.46	2.51 ± 0.50	0.083
TBA	-0.35 ± 0.81	-0.42 ± 0.77	-0.19 ± 0.88	0.029
**Arterial blood gas**				
PCO_2	3.52 ± 0.18	3.51 ± 0.17	3.54 ± 0.19	0.159
PO_2	4.91 ± 0.43	4.93 ± 0.42	4.85 ± 0.47	0.175
Intubated PO_2/FiO_2 ratio	5.80 ± 0.45	5.82 ± 0.44	5.74 ± 0.48	0.152
Serum sodium	4.92 ± 0.07	4.92 ± 0.08	4.92 ± 0.03	0.887
Serum potassium	1.44 ± 0.15	1.42 ± 0.14	1.48 ± 0.16	<0.001
Serum calcium	0.15 ± 0.07	0.14 ± 0.08	0.16 ± 0.05	0.002
Serum magnesium	-0.40 ± 0.26	-0.41 ± 0.30	-0.37 ± 0.17	0.102
Lactic acid	0.57 ± 0.59	0.51 ± 0.58	0.70 ± 0.60	0.011
HCO3-	3.03 ± 0.13	3.03 ± 0.13	3.02 ± 0.13	0.765
**Blood cell analysis**				
WBC count	2.53 ± 0.32	2.53 ± 0.33	2.54 ± 0.30	0.723
Lymphocyte count	-0.59 ± 0.48	-0.57 ± 0.48	-0.63 ± 0.48	0.332
Monocyte count	-0.09 ± 0.47	-0.10 ± 0.48	-0.08 ± 0.45	0.838
Neutrophil count	2.39 ± 0.30	2.38 ± 0.30	2.41 ± 0.31	0.463
RBC count	1.21 ± 0.21	1.21 ± 0.16	1.21 ± 0.30	0.993
Hemoglobin	4.61 ± 0.15	4.61 ± 0.15	4.59 ± 0.15	0.276
Hematocrit	3.43 ± 0.18	3.44 ± 0.19	3.41 ± 0.15	0.154
RDW	2.60 ± 0.10	2.60 ± 0.10	2.61 ± 0.09	0.795
Platelet count	4.93 ± 0.35	4.94 ± 0.31	4.90 ± 0.42	0.372
MPV	2.46 ± 0.11	2.45 ± 0.09	2.48 ± 0.15	0.093
PDW	2.68 ± 0.17	2.67 ± 0.17	2.69 ± 0.18	0.288
**Coagulation function**				

PT	2.51 ± 0.08	2.51 ± 0.08	2.50 ± 0.07	0.628
INR	0.07 ± 0.08	0.07 ± 0.08	0.06 ± 0.07	0.625
APTT	3.13 ± 0.84	3.16 ± 0.78	3.04 ± 0.97	0.294
Fibrinogen	1.04 ± 0.32	1.04 ± 0.29	1.02 ± 0.37	0.564
D-Dimer	0.19 ± 0.74	0.23 ± 0.68	0.10 ± 0.87	0.194

All variables are $\log_e$ transformed and present as mean ± SD. AKI, acute kidney injury; sST2, soluble ST2; NT-proBNP, N terminal pro-brain natriuretic peptide; H-FABP, heart-type fatty acid-binding protein; ALT, alanine aminotransferase; AST, aspartate transaminase; LDH, lactic dehydrogenase; UA, uric acid; HDL, high density lipoprotein; LDL, low density lipoprotein; TBA, total bile acid; WBC, white blood cell; RBC, red blood cell; RDW, red blood cell distribution width; MPV, mean platelet volume; PDW, platelet distribution width; PT, prothrombin time; INR, international normalized ratio; APTT, activated partial thromboplastin time.

Supplementary Table 2 Percentage of missing variables for each predictor in discovery cohort

biomarkers	Non-AKI(n=337)	AKI(n=115)
sST2	3.1%	3.4%
NT-proBNP	4.6%	6.9%
H-FABP	4.5%	1.7%
LDH	1.2%	4.3%
UA	0.3%	0.9%
